# A Goal Direction Signal in the Human Entorhinal/Subicular Region

**DOI:** 10.1016/j.cub.2014.11.001

**Published:** 2015-01-05

**Authors:** Martin J. Chadwick, Amy E.J. Jolly, Doran P. Amos, Demis Hassabis, Hugo J. Spiers

**Affiliations:** 1 Institute of Behavioural Neuroscience, Department of Experimental Psychology, Division of Psychology and Language Sciences, University College London, 26 Bedford Way, London WC1H 0AP, UK; 2 Clinic for Neurology, Otto-von-Guericke University, 39120 Magdeburg, Germany; 3 Gatsby Computational Neuroscience Unit, University College, 17 Queen Square, Alexandra House, London WC1N 3AR, UK

## Abstract

Navigating to a safe place, such as a home or nest, is a fundamental behavior for all complex animals. Determining the direction to such goals is a crucial first step in navigation. Surprisingly, little is known about how or where in the brain this “goal direction signal” is represented. In mammals, “head-direction cells” are thought to support this process, but despite 30 years of research, no evidence for a goal direction representation has been reported [1, 2]. Here, we used fMRI to record neural activity while participants made goal direction judgments based on a previously learned virtual environment. We applied multivoxel pattern analysis [3–5] to these data and found that the human entorhinal/subicular region contains a neural representation of intended goal direction. Furthermore, the neural pattern expressed for a given goal direction matched the pattern expressed when simply facing that same direction. This suggests the existence of a shared neural representation of both goal and facing direction. We argue that this reflects a mechanism based on head-direction populations that simulate future goal directions during route planning [6]. Our data further revealed that the strength of direction information predicts performance. Finally, we found a dissociation between this geocentric information in the entorhinal/subicular region and egocentric direction information in the precuneus.

## Highlights

• Virtual reality and fMRI were used to study human navigation
• The entorhinal/subicular region represents the geocentric direction to the goal
• The precuneus represents the egocentric direction to the goal

## Graphical Abstract

**Figure:**
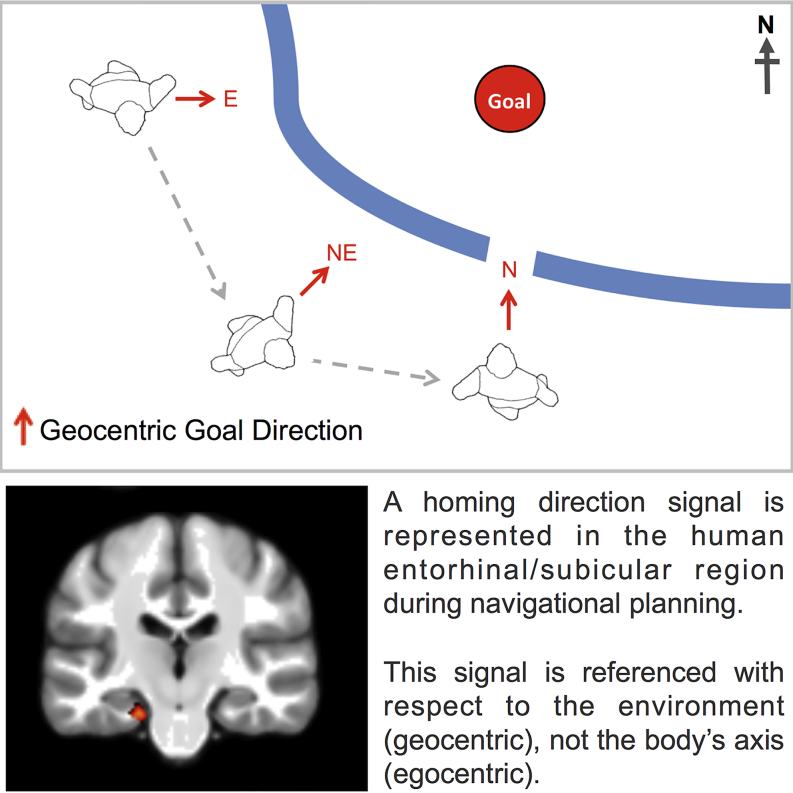

## Teaser

Chadwick et al. report evidence for a homing direction signal in the human brain. In the entorhinal/subicular region of the brain, this signal is relative to the axis of the environment (e.g., 45° northeast), and in parietal cortex, it is relative to the body’s axis (e.g., 45° to the left).

## Results

Navigating to a remembered goal requires knowing both your current facing direction and the direction to your goal. “Head-direction cells,” which fire when an animal is facing in a specific direction within the environment (e.g., north), have come to dominate models of how the mammalian brain represents direction information for navigation [1]. However, despite 30 years of research into direction coding, no neural representation of intended future goal direction has been discovered yet within the mammalian brain [2]. Models disagree as to where and how such a signal might be generated [6–10]. One possibility is that route planning may involve the simulation of the intended future route via transient recruitment of spatial representations active during travel to the goal [6, 8–13]. For example, simulation of an intended route would involve the sequential activation of place cells that represent locations along that future route. This influential theory has had support from recent findings that a rat’s future route can be predicted from the preactivation of sequential place cell firing within the hippocampus [14, 15]. Thus, the evidence to date supports the idea that future routes may be simulated by sequential place cell firing. However, models suggest that planning the route would also involve simulation of the direction to the intended goal. Indeed, such directional simulation may form the crucial initial stage of route planning [6, 8, 9], prior to subsequent place sequence activity. According to this proposal, during goal direction simulation, the head-direction cell population activity would change from cells responsive to current facing direction being suppressed to cells representing the desired future heading direction becoming active. Despite this clear theoretical prediction, the existence of directional simulation has yet to be empirically demonstrated in the mammalian brain. Here, we aimed to directly test for the presence of head-direction simulation within the human brain during navigational decision-making.

**Figure 1:**
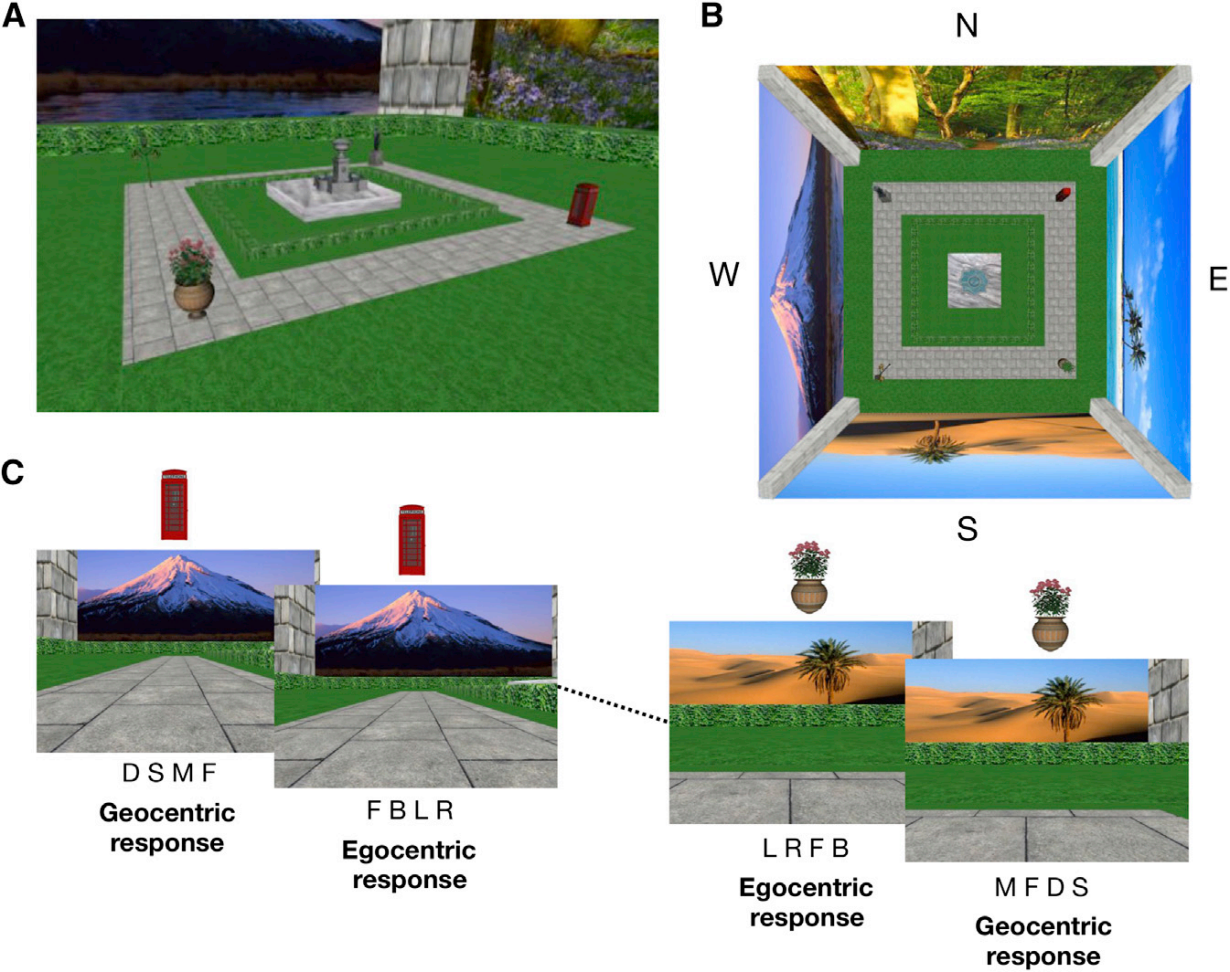
The Experimental Design (A) The layout of the virtual environment from an elevated view. The four key objects are visible, as are two of the distal scenes. Note that the participants never viewed the environment from this view but instead could only explore from ground level. (B) The same environment from an overhead, schematic view (not to scale). The four distal walls have been tilted so that they are visible from above. For clarity, we arbitrarily refer to the four cardinal directions as NSEW, but note that that they were never referred to as such during the actual experiment. (C) The goal direction task on two consecutive trials. The task was to judge the direction of the goal from the start location, and this could be required in one of two directional coordinate systems: environment-centered (geocentric) or body-centered (egocentric). For the geocentric question, participants were asked to decide which of the four distal scenes the goal location was toward from their start location (i.e., if they were to draw an arrow between the start and goal locations, which scene would it be pointing toward?). Although the focus of this study was on geocentric direction coding, we also included an egocentric question, in which the participant was asked to decide whether the goal location was located to the left, right, forward, or backward from the start location. Both the geocentric and egocentric questions were asked in every trial, with the order randomized. The four letters underneath each scene represent the four possible responses: in the geocentric task, these were desert (D), sea (S), mountain (M), or forest (F), which acted as semantic labels for the four cardinal directions. In the egocentric task, these were forward (F), backward (B), left (L), or right (R). The mapping between the four responses and the four buttons was partially randomized.

We applied multivoxel pattern analysis (MVPA) to fMRI data collected while participants (n = 16) made a series of goal direction judgments. All subjects gave informed written consent in accordance with the local research ethics committee. MVPA has been shown to be sensitive to specific neural representations in various domains [3–5], including place coding [16], scene coding [17–19], and facing direction [20–22]. It is therefore plausible that this approach may be able to detect neural representations related to simulation of future goal heading. Prior to scanning, participants learned the spatial layout of a simple virtual environment (Figures 1A and 1B) by freely moving around within it. The environment consisted of four objects placed at the corners of four paths arranged in a square. Each of the four distant edges of the environment consisted of a distinct scene in order to clearly differentiate the four cardinal directions. During scanning, participants were required to make goal direction decisions based on their memory of this environment (Figure 1C). Very high performance levels (mean 97% accuracy) indicated that participants were successfully able to engage goal direction systems (for more details on experimental design and methods, see Supplemental Information available online).

**Figure 2:**
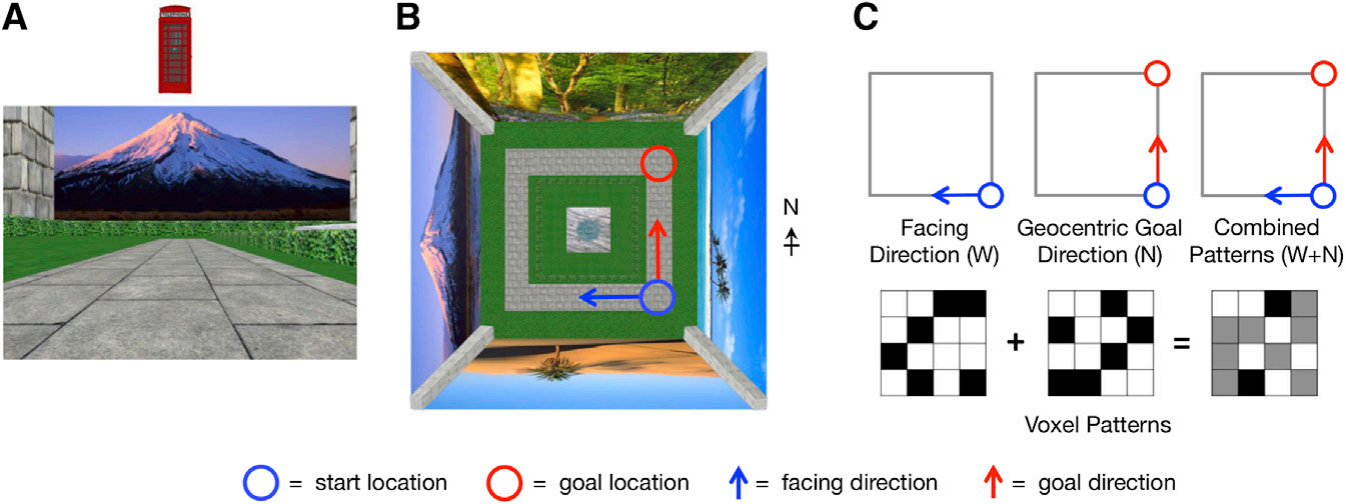
Each Experimental Trial Produces a Mixture of Both Facing Direction and Geocentric Goal Direction Information (A) Example of a single-decision trial, as presented to the participants. The scene displays the start view for the trial, and the object at the top represents the goal location for that trial. Based on this information, participants must first localize and orient themselves within the environment. Following this, they then calculate the direction to the goal. (B) An overhead schematic of the environment with start and goal locations displayed, along with facing and goal direction. (C) The top images show the direction information expected to be present over the course of this example trial if geocentric goal direction is represented via a process of simulation of future direction by head-direction populations. The bottom images display an abstracted representation of patterns of activity across 16 voxels (darker = greater activity) expected to be present during this same trial. Initially, the voxel pattern will represent current facing direction (West). During goal direction judgment, this head-direction population activity will shift to representing future geocentric goal direction (North), and the voxel pattern will shift accordingly. Due to the slow temporal resolution of the BOLD response, we detect a mixture of these two signals in a single trial. At the level of voxel activity, this equates to a mixture of the two patterns for West and North.

**Figure 3:**
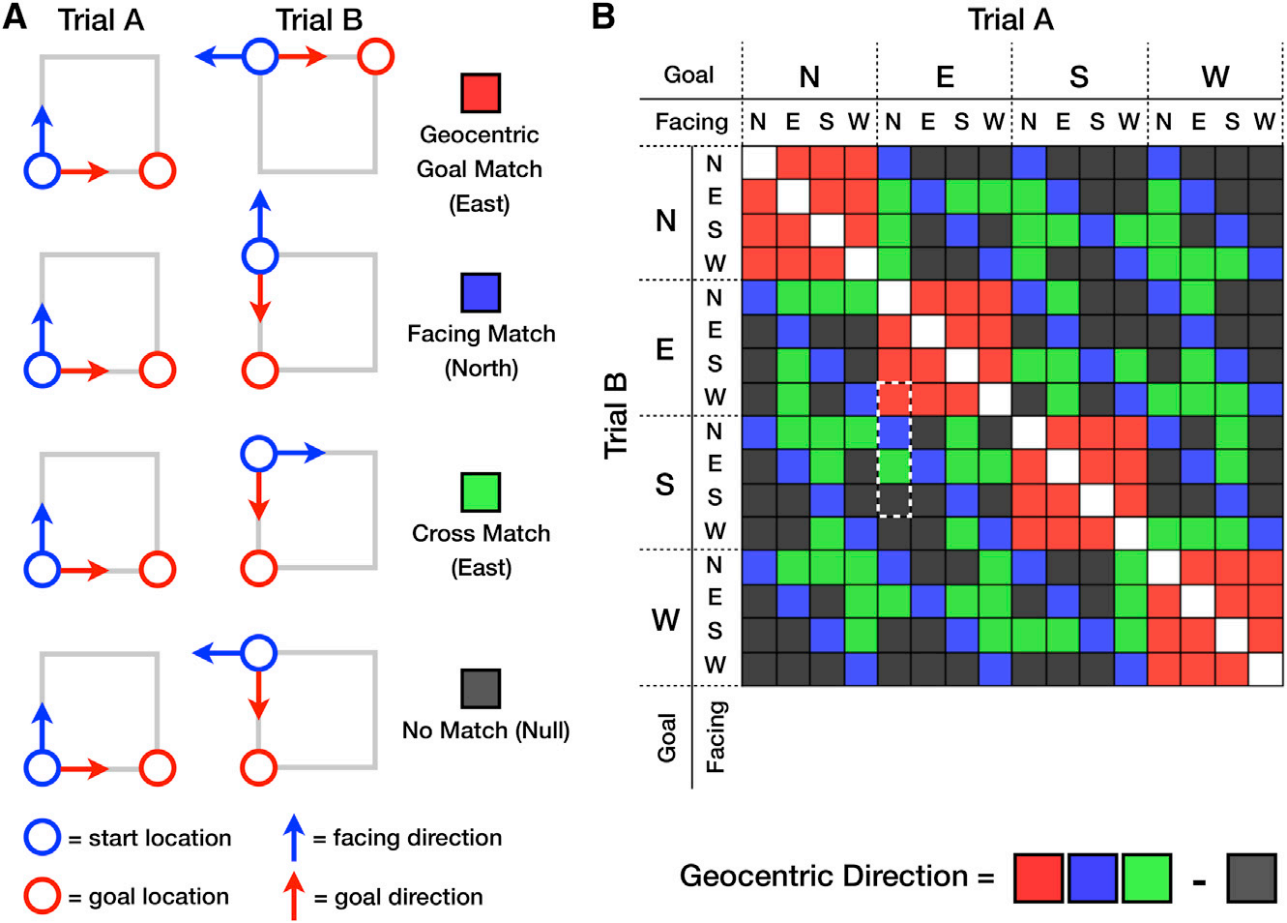
The Component Conditions Making Up the Geocentric Direction Analysis (A) Schematic pairwise match examples in four conditions (specific pairwise matches indicated by dotted white box in B). For each condition of interest, we provide an example trial pair where a specific direction matches, which therefore should show a higher pattern similarity than trial pairs that do not match (the null condition). The specific matching direction (e.g., North) is indicated in brackets underneath the match condition label. Each direction condition consists of the full set of trial pair matches fulfilling that condition. The cross match condition is particularly important for the head-direction simulation hypothesis, as this condition demonstrates a shared neural representation of geocentric direction regardless of whether the participant has a goal toward or is facing that direction. (B) Matrix representing the match condition for every possible pair of trials (although note that for simplicity, this is a reduced 16 × 16 matrix rather than the full 32 × 32 matrix. It nevertheless captures the key conditions). The labels along the top and left indicate the facing direction and geocentric goal direction of each trial. The color of each square indicates the match type (color codes provided in A). The white squares along the diagonal were excluded from the pattern similarity analyses (but see Supplemental Information for additional analyses related to these diagonal elements). Our general measure of geocentric direction information is derived through a contrast of the three geocentric conditions of interest (facing, goal, and cross match) against the null condition. The white dotted line indicates the four specific pairwise matches that are illustrated in (A).

Any region displaying head-direction simulation should contain two key neural representations: (1) current facing direction and (2) intended future goal direction. Furthermore, if the same neural population is involved in representing both current facing direction and simulating future goal direction, then we should find evidence for a single neural representation of each geocentric direction (e.g., north) that generalizes over both facing and goal directions. We assessed the representational code of a given region by investigating the pattern similarity between pairs of trials [23]. In the case of fMRI, pattern similarity was assessed by the spatial correlation in the blood-oxygen-level-dependent (BOLD) response between trial pairs. Trials that shared an underlying neural representation were expected to demonstrate greater similarity than those that did not. We expected to find a mixture of both facing and goal direction information present in each trial (Figure 2). Over the 32 distinct trials, the full combination of facing and goal directions was sampled, allowing us to separate these two types of information. Figure 3A displays the conditions used to infer both facing direction and goal direction independently. Importantly, we could also assess whether the neural pattern for a given geocentric direction (e.g., north) was expressed for both facing and goal directions, as we would expect if a head-direction population were recruited for the simulation of goal direction. To infer the presence of this kind of general direction information, we looked for increased pattern similarity between pairs of trials where the facing direction in trial A matched the goal direction in trial B, which we refer to as a “cross match” (Figure 3A).

We used a searchlight analysis [24] with multiple regressions [20] to search across the entire brain for regions displaying evidence of head-direction simulation while controlling for other salient factors, such as egocentric goal direction. A composite geocentric direction regressor was created by collapsing the three pattern similarity conditions of interest (facing, goal, and cross match) into a single binary regressor, contrasted against the pattern similarity expressed when none of these conditions were met (the null condition). As a head-direction simulation region should show increased pattern similarity under all three of these conditions, any such region should show a strong response to this geocentric direction regressor (Figure 3B). Statistical significance was assessed using nonparametric permutation testing [25]. Small-volume correction was applied based on strong a priori predictions about the neural regions involved in head-direction processing (Figure S1).

**Figure 4:**
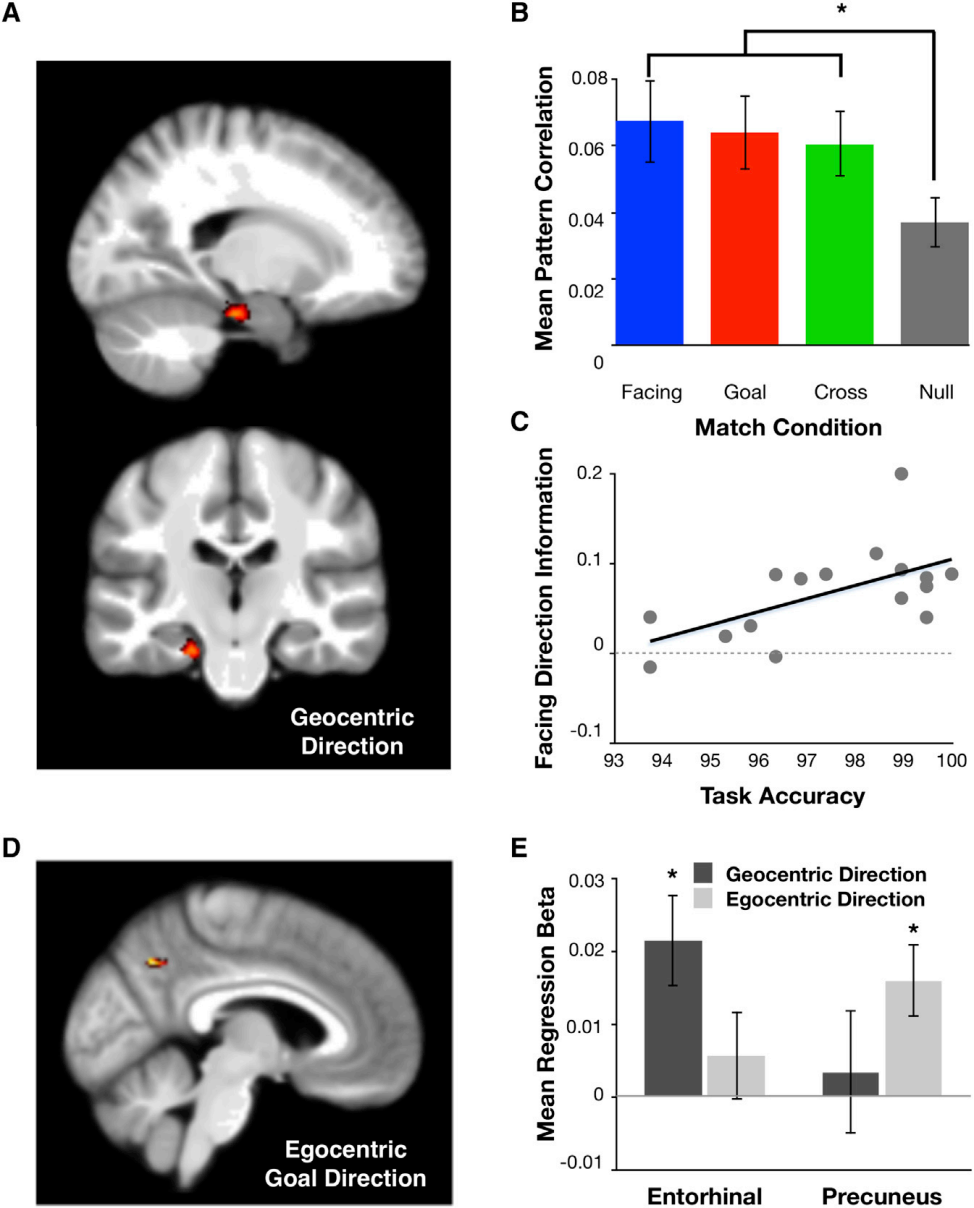
Geocentric Goal Direction Information Is Represented in Entorhinal/Subicular Region, while Egocentric Goal Direction Is Represented in the Precuneus (A) The geocentric direction searchlight analysis revealed a significant cluster (voxel threshold pseudo-t > 3, family-wise error [FWE]-corrected cluster threshold k > 88) in the left entorhinal/subicular region (peak Montreal Neurological Institute [MNI] coordinates: −20, −25, −24; peak pseudo-t = 3.8; cluster size = 157). This is displayed against the mean T1-weighted structural image. Although the searchlight result was left lateralized, further analyses demonstrated that the effect is bilateral in nature, with no evidence of a significant difference between the hemispheres (see Supplemental Information). (B) This region displays significant direction coding across all three conditions of interest. This was assessed by comparing the mean pattern similarity for each condition separately against the null condition, each displayed with standard error bars. (C) Individual variation in facing direction information within the entorhinal/subicular region correlates positively with goal direction task accuracy. (D) The egocentric direction searchlight analysis revealed a significant cluster (voxel threshold pseudo-t > 3, FWE-corrected cluster threshold k > 49) in the left precuneus (peak MNI coordinates: –6, –61, 39; peak pseudo-t = 3.3; cluster size = 50). This is displayed against the mean T1-weighted structural image. Despite the left lateralized peak of the precuneus result, further analyses suggest that precuneus represents this information bilaterally, with no hemispheric specialization (see Supplemental Information). (E) The neural coding of direction information type in the entorhinal/subicular region and precuneus was directly compared with a region-by-condition analysis. A significant interaction was found, which is consistent with a functional dissociation between the two regions, although it should be noted that neither region demonstrated a significant simple effect of condition. Nevertheless, the pattern of results is consistent with bias toward geocentric direction in the entorhinal/subicular region and egocentric direction in the precuneus, with the entorhinal/subicualr region coding for geocentric direction and precuneus coding for egocentric direction. Mean beta coefficients are displayed with standard error bars. See also Figure S1 for the small-volume correction ROIs used when applying this analysis; Figure S2 for a visual control analysis; and Figure S3 for example echo planar images demonstrating the extent of coverage within the entorhinal/subicular region.

This analysis revealed a significant effect centered on the left entorhinal cortex and extending into the presubiculum and parasubiculum (we refer to this as the entorhinal/subicular region). The effect was remarkably selective to this region (Figure 4A), with no other significant response anywhere else in the brain, even using a liberal statistical threshold (see Supplemental Information). Further investigation revealed that each of the three types of information was independently present (facing: t(15) = 3.48, p = 0.0017; goal: t(15) = 2.66, p = 0.009; cross: t(15) = 3.01, p = 0.0044). Thus, the entorhinal/subicular region contains all three individual components of a true head-direction simulation system (Figure 4B). Crucially, this includes a significant generalization of the neural pattern across facing and goal direction (the cross match condition), which provides clear evidence that the same neural populations are recruited for both facing and goal direction within a single trial. To further explore the nature of the entorhinal/subicular representations, we conducted a second analysis based on “pattern construction” [26–29]. This method uses a subset of the data to construct predicted neural patterns and then tests these predictions against the remaining data. This revealed that the voxel patterns expressed on any given trial were best explained by a linear mixture of both facing and goal direction pattern information (see Supplemental Information).

We next investigated the possibility that individual differences in the strength of the neural representations might influence task performance [30]. Such a relationship is a crucial element in demonstrating that such representations are directly relevant to navigational behavior, and yet such a relationship has not been demonstrated in previous studies of geocentric direction coding [20, 21, 31]. We found a significant positive correlation between entorhinal/subicular facing direction information and overall task accuracy (r(15) = 0.59, p = 0.016), which remained significant (r(15) = 0.64, p = 0.0095) after removing a possible outlier (defined using a cook’s distance threshold of 1). We also found a negative correlation between facing direction information and mean response time on the same decision task (r(15) = −0.56, p = 0.024). By contrast, goal direction information did not significantly correlate with either task accuracy (r(15) = 0.3, p = 0.27) or decision time (r(15) = −0.17, p = 0.52). These results therefore show that participants with a stronger representation of current heading direction are both more accurate and faster at making goal direction judgments in this task (Figure 4C).

In order to maximally differentiate the four cardinal directions within the virtual environment, we used four distinct distal scene cues. This raised the possibility that the results within the entorhinal/subicular region were driven purely by the visual properties of these four scenes. For example, if participants vividly imagined the intended route, then visual representations of the distal scene in the direction of the goal location might have been activated. If this is the case, then this could potentially explain the results in this region without requiring any abstract direction representation. In order to rule out this explanation, we included a visual control condition. This involved the presentation of the same set of start “views” but simply required the participant to categorize the displayed scene (e.g., forest), with no navigation requirements. This condition should activate purely visual neural representations, but not more abstract directional representations. Importantly, the entorhinal/subicular region did not contain any significant information about the visual scenes (t(15) = −0.53, p = 0.7), and visual scene information was significantly lower than geocentric direction information (t(15) = 3.28, p = 0.005). This demonstrates that this information is only present under conditions that require the computation of goal direction. By contrast, we looked within a region of interest in extrastriate cortex and found clear evidence of a neural representation of the visual scenes (t(15) = 1.83, p = 0.044). Indeed, a significant interaction (F(1,15) = 31.15, p = 0.0005) between pattern similarity condition (geocentric direction versus visual scenes) and region (entorhinal/subicular versus extrastriate) suggests the presence of a functional dissociation (see Figure S2), with the entorhinal/subicular region responsible for representing geocentric direction and extrastriate cortex responsible for processing the visual elements of the scenes. Neither main effect was significant. Consistent with this conclusion, a visual control version of the pattern construction analysis also failed to find any effect on the basis of visual scene information alone (see Supplemental Information).

While the main focus of this study was on head direction simulation and geocentric direction coding, our experimental design also allowed us to search for regions coding for egocentric goal direction. This revealed a selective result in the left precuneus (Figure 4D). Notably, the location of this result is consistent with the peak result of a previous MVPA study investigating egocentric direction coding [32], thereby providing a conceptual replication of the result. A further control demonstrated that egocentric information within the precuneus was not present while making egocentric judgments about dot locations on a screen (t(15) = −0.39, p = 0.65), and this information was significantly less than the navigation-based egocentric information (t(15) = 2.54, p = 0.023). This therefore suggests that the information contained within the precuneus is specifically related to navigationally salient egocentric information, supporting the purported role of medial parietal cortex in spatial navigation [2].

Thus far, our results have implicated two regions in the computation of goal direction. However, each of these regions appears to compute direction in a distinct coordinate framework: geocentric in the entorhinal/subicular region and egocentric in the precuneus. We further investigated the selectivity of these results and found that the entorhinal/subicular region shows no evidence for egocentric direction representations (t(15) = 0.94, p = 0.18), while the precuneus shows no evidence for geocentric direction representations (t(15) = 0.41, p = 0.34). To more formally test this apparent computational dissociation, we directly compared the two types of information (geocentric and egocentric goal direction) across the two regions of interest (ROIs) with a two-way repeated-measures ANOVA. Neither main effect was significant, but we did find a significant interaction (F(1,15) = 7.7, p = 0.014), which was clearly driven by a bias toward geocentric processing in the entorhinal/subicular region, and egocentric in the precuneus (Figure 4E). This pattern of results is consistent with theoretical models of navigation [8, 9]. For additional analyses of start and goal location representations, see Supplemental Information.

## Discussion

In summary, we show that the human entorhinal/subicular region supports a neural representation of geocentric goal direction. We further show that goal direction shares a common neural representation with facing direction. This suggests that head-direction populations within the entorhinal/subicular region are recruited for the simulation of the direction to future goals. These results not only provide the first evidence for the presence of goal direction representations within the mammalian brain but also suggest a specific mechanism for the computation of this neural signal, based on simulation. Furthermore, the strength of the entorhinal/subicular direction representation predicts individual variation in performance on our navigation task, showing that these computations play a direct role in active spatial decision-making. Finally, we find a dissociation between the environment centric representations in the entorhinal/subicular region and egocentric direction representations in the precuneus. Although this computational division of labor is predicted by various theoretical accounts of navigation [8, 9], this is the first study to demonstrate that both of these direction coordinate frameworks are active at the same time, presumably acting together in order to translate stored representations into representations for action.

The human entorhinal/subicular region has previously been shown to contain grid cells [33, 34], cells coding direction of motion [35] and representations of distance to a goal [36, 37]. In rodents, it is also known to contain conjunctive grid cells, which are modulated by heading direction [35]. Although it is not clear how our results could be due to the activity of classical grid cells, we cannot rule out a possible contribution from conjunctive cell ensembles simulating intended future vectors [9]. However, such a process is unlikely to occur in the absence of direction simulation within the connected head-direction populations (see Supplemental Information for an extended discussion). We therefore suggest that head-direction simulation remains the most parsimonious explanation for our results.

Due to the relatively poor temporal resolution of fMRI, we are not able to determine what the temporal dynamics of head-direction simulation may be. Our assumption is that head-direction populations are initially involved in representing current facing direction and then switch to simulation during navigational planning. However, other temporal dynamics, such as constant oscillation between facing and goal direction, would explain our results equally well. Thus, we remain agnostic regarding the precise temporal dynamics involved in head-direction simulation, which will have to be resolved with alternative methodological approaches.

Given the involvement of the medial temporal lobe in episodic memory [38], we considered whether our data could be interpreted in terms of episodic memory rather than direction coding per se. Importantly, our results provide evidence for direction coding that generalizes across different locations. If episodic memory were the only process involved, we would expect to find a unique neural representation for each combination of location and direction and would not find evidence for direction coding that generalizes across locations. Therefore, we do not believe that episodic memory alone can explain our data, although it may have aided the retrieval of the geocentric direction representations. We can similarly rule out an account based on associative encoding of each triad of start location, goal location, and geocentric direction. This is due to the fact that learning involved free exploration of the environment rather than explicit exposure to each of the 32 possible associative triads (see Supplemental Information).

One limitation of the current study was the use of a standard 3 mm^3^ voxel resolution. Although this was necessary in order to allow us to explore information across the whole brain, it did constrain our ability to determine the precise anatomical region providing the goal direction signal [39]. In particular, both the entorhinal cortex and presubiculum are clear candidates for such a signal, but given their small size and close proximity, smaller voxel resolution would be required to accurately separate these two regions [40]. Further research at higher resolution will be needed to accurately determine which precise region is contributing to goal direction.

Overall, our results provide important new insights into the neural circuits involved in computing the direction to a desired goal beyond the current field of view and suggest that simulation within the entorhinal/subicular region may play an important role. Future work will need to confirm that simulation is indeed the key mechanism underlying these results, as well as demonstrating a causal influence of this mechanism on spatial cognition. Furthermore, it will be important to demonstrate that such mechanisms extend beyond the kind of simple environment used in the current study, into larger and more complex environments. Electrophysiological investigations of head-direction neuronal populations within the entorhinal/subicular region in rodents should allow more detailed investigation of these goal direction computations.

## Author Contributions

All authors were involved in designing the experiment. M.J.C. and A.E.J.J. collected the data. M.J.C. analyzed the data. M.J.C. and H.J.S. wrote the paper.

## Supplemental Information

Document S1. Supplemental Results, Supplemental Discussion, Supplemental Experimental Procedures, and Figures S1–S3

Document S2. Article plus Supplemental Information
