# Supplementary material for: A Goal Direction Signal in the Human Entorhinal/Subicular Region
Source: Curr Biol. 2015 Jan 5;25(1):87–92. doi: 10.1016/j.cub.2014.11.001 (PMC4291144; doi:10.1016/j.cub.2014.11.001)
Supplement: Document S1. Supplemental Results, Supplemental Discussion, Supplemental Experimental Procedures, and Figures S1–S3 [file mmc1.pdf]

Current Biology, Volume *25*

Supplemental Information

A Goal Direction Signal in the Human Entorhinal/Subicular Region

Martin J. Chadwick, Amy E.J. Jolly, Doran P. Amos, Demis Hassabis, and Hugo J. Spiers

Supplemental Figures

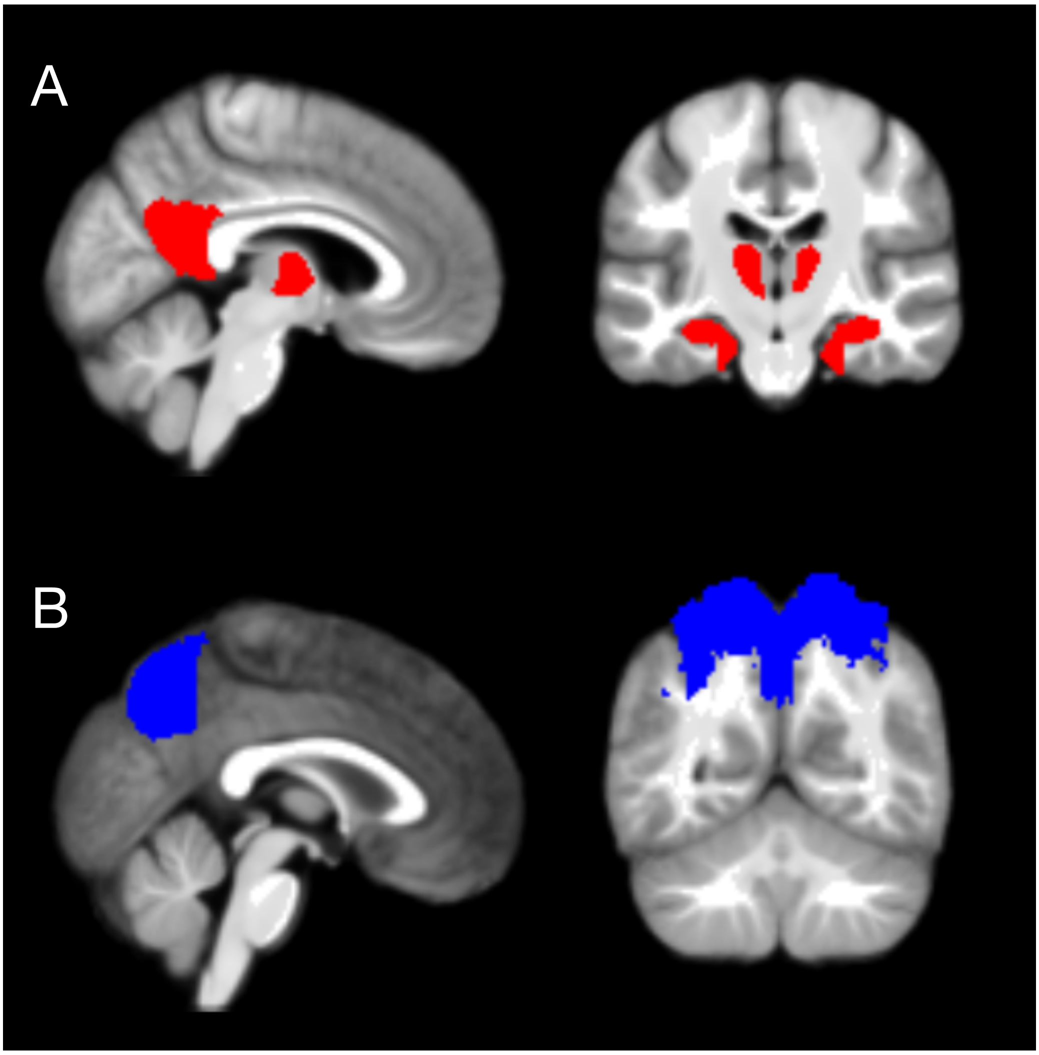

Figure S1, related to Figure 4. The regions-of-interest used for small-volume correction

(A) A bilateral set of regions thought to be important for head- direction coding was combined into a single mask for the geocentric direction searchlight small-volume-correction. This included the hippocampus, subiculum, entorhinal cortex, retrosplenial cortex, posterior cingulate cortex, and a portion of the thalamus including anterior and lateral dorsal regions. (B) A bilateral set of parietal regions thought to be important for egocentric direction coding was combined into a single mask for the egocentric direction searchlight small-volume-correction. This included the intraparietal sulcus, Brodmann area 7, and the precuneus.

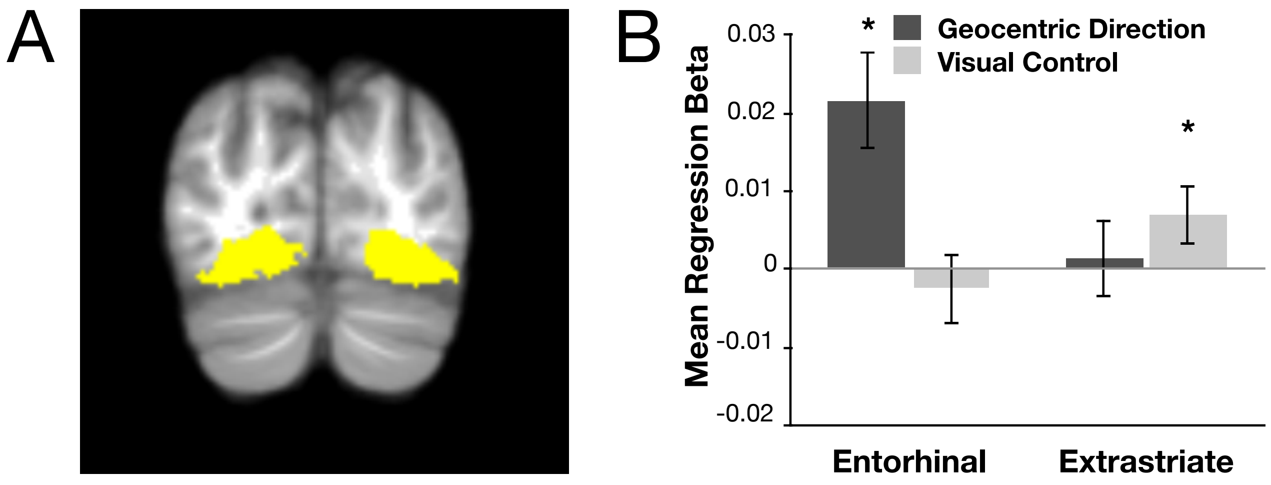

Figure S2, related to Figure 4. Dissociation between visual cortex and entorhinal/subicular region

(A) Visual scene processing in the extrastriate cortex was investigated with a bilateral anatomical ROI. (B) The entorhinal/subicular region does not contain visual information about the four distal scene cues. By contrast, extrastriate displays significant coding of visual scene information. A region by information-type ANOVA revealed a significant interaction effect, suggesting a double-dissociation between the neural coding in these two regions, with the entorhinal/subicular region selectively coding for geocentric direction. However, we note that while the entorhinal/subicular region displays the expected simple effect ($t(15)=3.34$, $p=0.005$), the extrastriate cortex simple effect was not significant ($t(15)=0.98$, $p=0.39$). Thus, while the pattern of results is consistent with a functional double dissociation, the data do not conclusively demonstrate this. Nevertheless, our data do firmly support the conclusion that visual scene information is not present within the entorhinal/subicular region, which was the main purpose of this control analysis. Standard error bars are displayed.

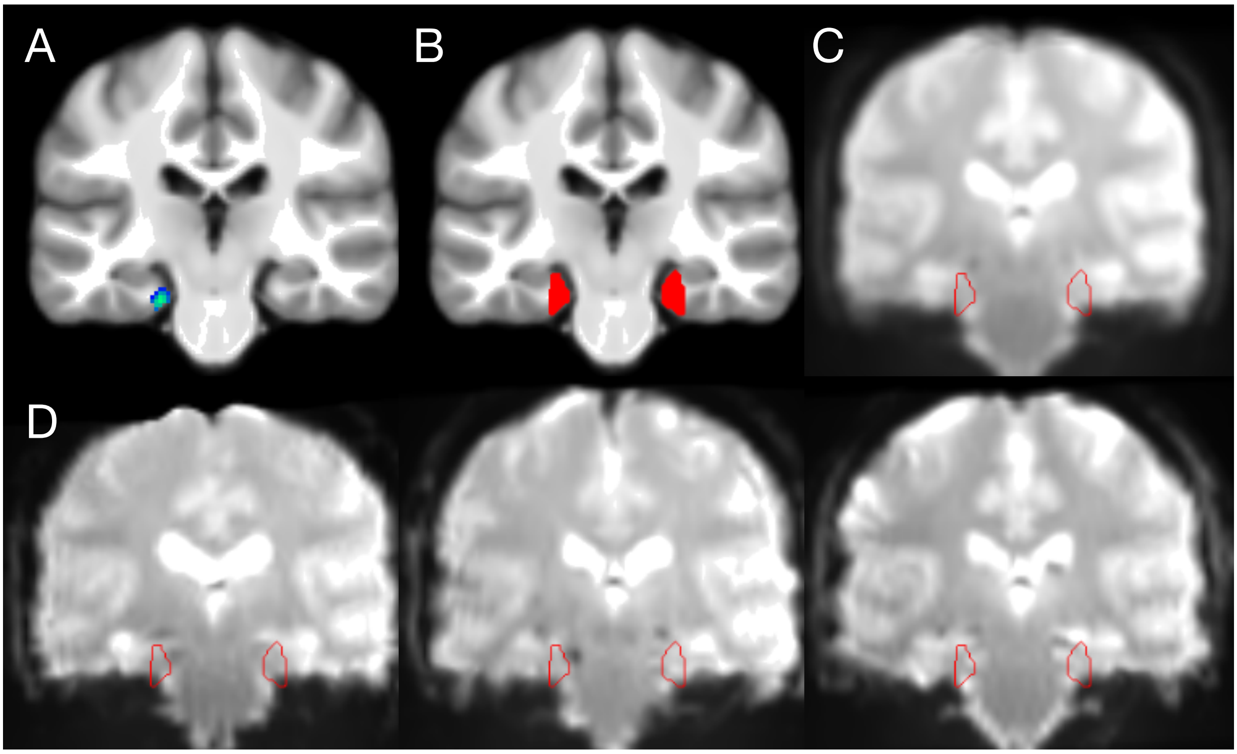

Figure S3, related to Figure 4. BOLD signal in the entorhinal region.

(A) The result of the geocentric direction searchlight analysis, displayed against the group mean average structural image. The result is displayed on a coronal slice at y=-25, which contains the peak of the geocentric searchlight result. (B) The entorhinal portion of the structural mask used for small volume correction of the geocentric direction searchlight analysis. This is displayed against the group mean average structural. (C) The outline of the entorhinal mask is displayed against the group mean average EPI image, based on normalized, unsmoothed data. While signal dropout is apparent in more ventral portions of the medial temporal lobe, the region within the entorhinal mask still displays a good signal (D) The outline of the entorhinal mask is displayed against the mean EPI image for three example participants. These three participants were chosen in order to display the full range of tSNR signals (based on the functionally defined entorhinal ROI). The EPI series with the highest mean tSNR value of 52 (maximum voxel snr = 102, minimum = 15) is displayed on the left, the middle image has a mid-range mean tSNR of 41 (maximum = 82, minimum = 11), and the right image has the lowest mean tSNR of 29 (maximum = 62, minimum =

9). While there is variation in the extent of dropout in more ventral MTL, all three participants display minimal or no signal dropout within the entorhinal mask.

Supplemental Experimental Procedures

Participants

Sixteen healthy right-handed participants (7 female) were recruited from University of London institutions. All had normal or corrected-to-normal vision and gave written consent to participant in accordance with the local research ethics committee.

Virtual environment

This environment was created using the Vizard VR Software Toolkit. The environment consisted of four paths arranged in a square, with a goal object at each corner of the square (phone box, statue, lamppost, and flowerpot). At the four edges of the environment we placed four distinct scenes (forest, mountain, desert, sea) in order to provide clear, unambiguous information about the four cardinal directions of the environment. Effectively these four scenes provide a clear cue for environment-centred orientation, equivalent to North, South, East, and West. In the centre of the square was a fountain, to provide additional visual information for orientation, and at each of the corners, between the distal scenes, was a tall stone pillar, to provide clear separation of the different scenes. Similarly, the four paths were not functionally relevant to the task itself, but could provide additional visual information for orientation. The participants were not restricted to the paths during initial learning, but could freely explore the entire environment. This specific environment was created in order to create the simplest possible experimental design that would allow us to investigate goal direction information. Four objects arranged in a square is the minimum spatial arrangement required so that there are two potential goal-direction vectors in

each of four cardinal directions, thereby allowing us to look at generalized representation of direction across the specific vectors. By minimising the simplicity of the design, we were able to maximise the number of trials within each condition, thereby maximising the power of our analysis.

Using VR to investigate navigation

Under normal circumstances, both visual and vestibular inputs play an important role in determining the activity of head-direction populations. However, when navigating within a virtual environment, the vestibular input will be uninformative for guiding navigation. Given this, is virtual reality a viable method for investigating head direction representations [S1]? We suggest that it is reasonable to assume that, in the absence of informative vestibular input, head-direction neurons will be dominated by visual information instead. This assumption is supported by previous results indicating that reliable place and grid cell activity can be detected in humans while they navigate within a virtual environment, where vestibular input is uninformative [S2–S5]. We think it unlikely that these specialized spatial neurons would be active in isolation from any input from head-direction cells. Indeed, Doeller et al. [S4] used extensive modelling of their data, based on electrophysiological recordings in the rat entorhinal cortex, to demonstrate that their results depend on intact conjunctive grid cell populations. Given that conjunctive cells receive their direction input from head-direction cells [S6, S7], this result provides evidence that head-direction signals remain intact within a virtual environment. Furthermore, facing direction coding has been found within human presubiculum and retrosplenial cortex [S8]. These results fit very well with the known location of head direction populations. Put together, there is now strong evidence to suggest that virtual reality taps into the same spatial mechanisms as during real-world navigation, including head-direction populations.

Experimental design

Prior to scanning, the participants learned the spatial layout of a simple virtual environment (Figures 1A and 1B) through twenty minutes of free exploration. While they explored this environment, participants were instructed to pay particular attention to the location of the four goal objects, as we would later be testing their memory for this information. To aid our description of the experiment, we will subsequently refer to these cardinal directions as NSEW, based on our own arbitrary labels of the layout (see Figure 1B), but note that we never referred to compass directions during the actual experiment, and never asked the participants to refer to compass directions. Interspersed with free exploration time, we presented eight probe trials to the participant to ensure that they were learning the environment. These probe trials were identical to the goal direction questions in the main scanning task, described below. After the learning session, but prior to entering the scanner, the participants completed one block of the full experiment in order to ensure they were well practised at the navigation task, and to try and minimize any effects of learning during scanning.

The structure of a single trial of the task is shown in Figure 1C. There were four components to each trial: first there were two control tasks, then two varieties of goal direction task. First we describe the goal direction tasks, before explaining the purpose of the control tasks. For the goal direction tasks, the participant was presented with a view taken from within the virtual environment (Figure 1C). This view was always taken from one of the four goal object locations, but with all objects now removed from the environment. The view was in one of the four cardinal directions within the environment, and the pictures were cropped to ensure that they included only the distal scene that was directly in front of them, and not any of the other scenes. This picture indicated the "start location" for the trial, and the participant had to determine exactly

where this view was located within the environment, and which direction they would be facing in, based on the depicted view. Above this view, a picture of one of the four goal objects was presented against a white background. Participants were instructed to recall where this object was located within the environment, and this acted as the “goal location” for the trial. The goal location was never at the start location, and was also never located diagonally across from the start location – it was always directly towards one of the cardinal directions from the start. The task was to judge the direction the goal was from the given start location, and this could be one of two types of direction: environment-centred (geocentric) or body-centred (egocentric). For the geocentric question, participants were asked to decide which of the four distal scenes the goal location was toward from their start location (i.e. if they were to draw an arrow between the start and goal locations, which scene would it be pointing towards?). This choice was made by pressing a button corresponding to the scene label (the first letter of the corresponding scene) underneath the start view. While the focus of this study was on geocentric direction coding, we also included an egocentric question, where the participant was asked to decide whether the goal location was located to the left, right, forward, or backward from the start location. Importantly, as we could independently manipulate start location, start view, and goal location, we were able to decorrelate geocentric and egocentric goal direction over trials. The two goal direction questions (geocentric and egocentric) were both presented on every trial, and the order of the questions was randomized across trials. Each of the four tasks on each trial was self-paced, and there was a 1s inter-stimulus interval between each trial.

On each trial, prior to these goal direction tasks, there were two control tasks: a geocentric control and an egocentric control. The geocentric control involved the presentation of the same start view that would be used in the later goal direction tasks. However, no goal object was presented, and the task was simply to name the scene category featured in the view by pressing a button

corresponding to the scene label. This task therefore involved visual and semantic representations of the distal scene cues, but did not require any navigation processing. This condition allowed us to ensure that any geocentric directional information was not simply based on visual or semantic representations of the distal scenes. The egocentric control also featured this same view, but in addition, a red dot was presented on this scene. The task was to decide whether the dot was to the left, right, above, or below the centre of the scene. This task therefore involves egocentric spatial decisions that are not navigationally relevant. These two control tasks were both presented on every trial, and were always presented prior to the goal direction tasks. The order of the two control tasks was randomized across trials. Each task block included 32 trials (which comprised the combination of each start location, start view, and goal location) presented in a random order. The participants completed three task blocks in the scanner, each collected in a separate functional run.

While the response to each component of each trial was self-paced, we report the mean response times for each of the four tasks:

Geocentric goal direction – 4.52s (sd = 1.41s)

Egocentric goal direction – 3.58s (sd = 0.86s)

Geocentric control – 2.30s (sd = 0.75s)

Egocentric control – 1.91s (sd = 0.41s)

Participants all had very high levels of accuracy in both goal direction tasks (geocentric mean accuracy = 97% [sd = 0.02]; egocentric mean accuracy = 97% [sd = 0.02]), and in both control tasks (geocentric control mean accuracy = 99% [sd = 0.01]; egocentric control mean accuracy = 98% [sd = 0.02]), demonstrating that they had learned the virtual environment very well. Notably, despite

the fact that all participants performed near ceiling on both tasks, there was nevertheless adequate variance to detect a strong positive correlation between geocentric and egocentric task performance ($r(15) = 0.77$, $p = 0.00047$). This suggests that the individual variance in both decision tasks may be due to common cognitive and neural components. These two scores were therefore pooled for the purposes of investigating the neural correlates of overall navigation performance.

MRI scan details

All MRI data was collected using a Siemens Avanto 1.5 tesla MRI scanner with a 32-channel head coil at the Birkbeck-UCL Centre for Neuroimaging (BUCNI) in London. The functional data was acquired using a sequence that has been optimised for medial temporal lobe coverage by the physics team at BUCNI. This was a gradient-echo EPI sequence in an ascending sequence, with a slice thickness of 2mm and a 1mm gap, TR=85ms, TE=50ms, slice tilt=-30°, field of view 192mm, and matrix size 64x64. The whole brain was acquired with 40 slices, leading to a volume acquisition time of 3.4s. The precise slice tilt was chosen as a compromise between sensitivity, coverage, and speed [S9, S10]. Three functional runs were collected for each participant. Following functional imaging, an anatomical image was acquired for each participant (T1-weighted FLASH, TR = 12ms, TE = 5.6ms, 1mm^3 resolution).

Imaging the entorhinal region

It is well known that parts of the medial temporal lobe are susceptible to BOLD signal dropout, making it a difficult region in which to reliably detect effects of interest [S11]. In order to reduce such problems, we used a scan sequence that was optimized to minimize signal dropout. In particular, both thin acquisition slices and slice tilting have been shown to reduce dropout [S9, S10]. As displayed in Fig S3, the scan sequence used in this study was able to detect a reliable BOLD signal within the entorhinal region despite some signal dropout in the ventral portions of the

medial temporal lobes. We further investigated the strength of the entorhinal BOLD signal by calculating the temporal signal-to-noise ratio (tSNR) within our functionally defined entorhinal/subicular region of interest [S12]. This was calculated from the normalized, unsmoothed EPI images. For each session separately, the voxel-wise tSNR was calculated as the mean/standard deviation of each voxel time-series. Then for each subject the three sessions were averaged to create a single summary tSNR value at each voxel within the ROI. We calculated the mean tSNR across voxels, as well as the maximum and minimum voxel tSNR for each subject. At the group level, the overall mean tSNR was 43.3 (sd = 6.7), indicating that a BOLD signal was reliably present within this region. The mean of the maximum tSNR was 89.3 (sd = 12.0), and the minimum was 9.9 (sd = 3.0), which demonstrates that there was substantial variation in tSNR across voxels within the ROI.

fMRI pre-processing

The first six functional volumes were discarded to allow for T1 equilibration. The remaining functional volumes were spatially realigned to the first image of the first functional series. Each participant's structural image was co-registered to the first functional image. The structural images were segmented and normalized to the DARTEL template using the VBM8 toolbox. The warps from this procedure were then applied to the functional data. This procedure normalized all MRI data into MNI space. Note that this procedure also resliced the functional voxels into a new interpolated resolution of $1.5mm^3$. For the main analyses, each trial-type (unique start-view-goal combination) was modelled with a different regressor including its instance in each of the three functional runs. Each trial was modelled with a boxcar regressor which started from the response to the first goal-direction question, and ended at the second response. The reasoning was that both geocentric and egocentric goal direction information should be required regardless of the type of goal direction response required, and so the specific modality of explicit goal response was

not differentiated in these models. Furthermore, we expected the information to be maximally present at, or just before the first goal direction decision, and to continue to be strongly represented up until the second decision. We therefore elected to model this specific period of time during the trial. This provided a parameter estimate at each voxel for each of the 32 trial types. These were converted to t-statistics by dividing the parameter estimate by the estimate of the standard error, thereby normalizing the responses of each voxel [S13, S14]. For each control analysis, the same general approach was used, with the difference that each unique trial-type was modelled using an event-related regressor locked to the time of the responses, rather than a boxcar regressor. The resulting t-statistic images were left unsmoothed to preserve any fine-grained spatial information [S14].

Small-volume correction regions-of-interest

For the purposes of hypothesis-driven small-volume correction of the searchlight analyses, we created two anatomical masks. The first was for the geocentric direction analysis, and consisted of the combination of a bilateral network of regions thought to contain head direction neuronal populations. This included the hippocampus [S15] and entorhinal region [S16], retrosplenial and posterior cingulate cortex [S17], and a region of the thalamus approximately covering the anterior and lateral dorsal nuclei. This mask was created by manually delineating each of these regions on the average T1-weighted structural image, and then combining all regions into a single mask. As many of these regions have also been implicated in coding for spatial location within an environment [S3, S8, S18], we used this same small-volume mask for the analysis of start location information. The second small-volume mask was for the egocentric direction analysis, and consisted of the intraparietal sulcus (IPS) and precuneus bilaterally. This mask was based on a combination of regions BA7 and the IPS taken from the SPM Anatomy toolbox [S19], but was

manually adjusted to include a greater extent of the precuneus [S20]. See Figure S1 for images of each of these ROIs.

Searchlight pattern similarity analysis

We used a searchlight analysis in order to search across the whole brain for regions containing specific neural representations [S21]. This involves stepping through each voxel in the brain, and in each case running a pattern similarity analysis on the cluster of voxels surrounding that central voxel (for all analyses, we used a 10mm radius spherical searchlight). This provides a measure of information at every single voxel, and a group-level statistical analysis can determine whether there are any regions containing a significant amount of information across the group of participants. For the pattern similarity analysis itself, the similarity between each pair of trials was measured using a Pearson correlation between the patterns of fMRI voxel activation within the ROI across each pair of trials. This results in a similarity matrix, with each point in the matrix representing the pattern similarity between a pair of trials. For the primary analyses we used a multiple binary regression approach [S8]. Each regressor specifies a contrast of one part of the similarity matrix against the rest (Figure 3B). By using this approach, it is possible to test the contrast of interest while controlling for variance that is attributable to other variables. The multiple regression included four regressors: geocentric direction, egocentric goal direction, start location, and goal location. Geocentric direction was our primary regressor of interest, and specified a combined contrast of three similarity conditions (facing, goal, and cross match) against the null condition (Figure 3A). Any region with head direction simulation properties should include all three of these conditions, and the geocentric direction regressor should therefore detect any such region. Egocentric goal direction specified all trial pairs where the egocentric direction to the goal was the same (e.g. left), against those where they did not. This was our regressor of interest for the secondary egocentric analyses. Start location specified all trial pairs where the start

location matched against those where they did not, and similarly, goal location specific all trials pairs where the specific goal location matched. While these additional regressors were not completely uncorrelated with the geocentric direction regressor, r^2 was very small in each case (0.01 for both start location and goal location, and 0.03 for egocentric direction), and well within tolerance limits for multiple regression analysis. Thus, this analysis fully controlled for these extraneous factors.

This searchlight pattern similarity multiple regression analysis results in a beta parameter estimate for each regressor at each voxel. The brain-wide set of parameter estimates for each participant was passed to a group-level nonparametric statistical analysis for each condition of interest (see next section). Small-volume correction was applied separately to each searchlight analysis, based on strong a priori predictions about the neural regions involved in processing each type of information (see above and Figure S1).

Nonparametric searchlight statistics

Parametric assumptions regarding the spatial distribution of data may not hold with unsmoothed data. Nonparametric statistical approaches do not make such assumptions, making them more suitable for use with unsmoothed data. We therefore analysed the group-level data using statistical nonparametric mapping [S22]. We used 10mm variance smoothing and 10,000 random permutations. A cluster-based threshold was used to assess statistical significance, using an initial cluster threshold of Pseudo-t = 3. Results were considered significant at a family-wise-error-corrected cluster threshold of $p<0.05$. This method of FWE correction proceeds as follows. Over 10,000 permutations, each searchlight beta image is randomly assigned to be multiplied by either 1 or -1. Under the null hypothesis, we expect that the set of beta values at each voxel should be symmetric about zero, and hence for a single subject, the observed data can be flipped without

altering its distribution. Thus, under the null hypothesis, randomly flipping the sign of a single subject's beta-map will leave the joint distribution unchanged. At each voxel, the pseudo-t statistic (more on this below) is calculated for both the observed data, and each set of permuted data. In order to apply cluster correction for multiple comparisons, an initial voxel-level threshold is applied to each dataset (in this case set to t=3, as suggested by [S22]) Based on this initial threshold, the permutation distribution of the maximal suprathreshold cluster size can be derived. This is accomplished by noting, for each permuted statistic image, the largest size of cluster above the initial voxel-level threshold. Corrected p-values for each suprathreshold cluster in the observed statistic image are obtained by comparing their size to the permutation distribution. By forming the null distribution from the maximal statistic in each permuted image, this ensures that any resulting p-values are fully corrected for multiple comparisons.

The use of nonparametric statistics in fMRI allows the use of variance smoothing, whereby the variance estimate at each voxel is based on a pooled estimate, spatially smoothed across neighbouring voxels. This new, pooled variance is then used to compute a more reliable statistic at that voxel. The resulting statistic does not have a Student's t-distribution under the null hypothesis, hence the term Pseudo-t statistic. Overall this procedure will somewhat increase the smoothness of the statistic image, but only through a reduction in high-frequency noise. Any fine-grained differences in signal between neighbouring voxels will be preserved, making this approach entirely suitable for MVPA analysis.

Regions-of-interest from the searchlight analyses

Following the searchlight analyses, we ran a series of follow-up analyses within each region. For each functional cluster (entorhinal/subicular region and precuneus), we defined the region of interest using a threshold of Pseudo-t > 2, in order to provide enough voxels for ROI-based RSA

analysis. Notably, at this more liberal threshold, the precuneus cluster is bilateral in nature. The bilateral extrastriate cortex ROI was defined using the SPM Anatomy toolbox [S19].

ROI statistical tests

For all ROI-based tests, each correlation coefficient was normalized using Fisher's r-to-z transformation, in order to allow parametric testing at the group level. Group-level statistics were performed on either the set of beta parameters or correlation coefficients from a given region or regions across participants. When determining whether a region contains significant information (i.e. testing beta parameters against zero, or testing correlation coefficients against the null match condition), one-tailed t-tests were used as recommended by current methods guidelines for pattern similarity analysis [S23]. The reason for this is that, given our experimental hypothesis, we expect an increase in pattern similarity in our experimental compared to null condition, but the same hypothesis does not provide any explanation for a significant drop in similarity compared to the null. Note that this is in contrast to a similar analysis of univariate data, where it is always possible that there could be a task-driven deactivation, meaning that two-tailed tests in this case are to be preferred. When comparing information across two conditions of interest (e.g. geocentric direction vs. visual scene information), or across two regions (e.g. entorhinal/subicular region vs. precuneus), two-tailed t-tests were used, as here a significant result could reasonably be expected in either direction.

Pattern similarity contrasts in the entorhinal/subicular region

The geocentric direction analysis used in the searchlight analysis consisted of the combination of three conditions (facing, goal, and cross match). In theory, any one of these conditions could have driven this response without the contribution of the other two – for example, the response could be driven purely by information about current facing direction, which we know to be present

within the presubiculum (which forms a part of our functional cluster) from previous work [S8]. To infer the existence of a genuine head direction simulation system, we need to demonstrate the presence of each of these three types of direction information independently. Within the entorhinal/subicular ROI we looked at each type of directional information separately, in each case contrasting the pattern similarity against the null condition using a one-tailed paired t-test. Note that any similarity pair that fell into more than one condition (e.g. facing and goal direction) was excluded, to ensure specificity within each condition. Furthermore, as we were specifically interested in directional information that generalizes across specific location, we also excluded similarity pairs that shared the same start location. Thus, any residual information could only represent a generalized direction representation. Significance was assessed using a Bonferroni-corrected α threshold of $p<0.0167$ to control for the three separate statistical tests.

Pattern similarity correlation with behavioural variables

We investigated correlations between both facing direction and goal direction entorhinal/subicular neural representations and individual variation in task accuracy. We particularly focussed on task performance, as a previous study has found that entorhinal processing predicts variation in navigation performance across individuals [S4]. Due to the high correlation between geocentric and egocentric task performance (see above), we used accuracy pooled across both tasks. A Pearson correlation was used in each analysis, and a significance threshold of 0.025 was used, Bonferroni-corrected for the two correlation analyses. Results are reported in the main text, but in brief, a significant positive correlation was found between accuracy and facing direction, but not goal direction. Task accuracy and reaction time were found to be highly correlated ($r(15)=-0.76$, $p=0.0007$), and so we predicted that we should find a similar pattern of results in the reaction time data as with accuracy – i.e. a correlation between reaction time and facing direction information, but not goal direction information. Given this clear

prediction, we used a significance threshold of $p<0.05$ to assess the correlation between facing direction information and reaction time (although we note that the result would also be significant using a more stringent $p<0.025$ threshold).

We repeated each of the correlation analyses with facing direction information based on the unpooled accuracy and reaction time data, in order to check that the results still held. These analyses found that facing direction information correlated significantly with egocentric task accuracy ($r(15)=0.64$, $p=0.008$) and reaction time ($r(15)=-0.53$, $p=0.035$), and trended to significance with geocentric task accuracy ($r(15)=0.46$, $p=0.07$) and reaction time ($r(15)=-0.49$ $p=0.055$). Thus, even using unpooled data, both tasks produced either significant results, or trended toward significance in the expected direction. We therefore conclude that pooling the behavioural data across these tasks did not make a substantial impact on the resulting data.

Visual control analysis

In the visual control condition of each trial, we presented the start view, but instead of requiring a navigation decision, participants simply had to categorize the displayed scene (Forest, Mountain, Desert, or Sea). This condition should activate purely visual neural representations, but not more abstract directional representations. Thus, this condition allowed us to search for the presence of purely visual information. Visual control data were preprocessed using a first-level GLM to fit a regressor for each of the 32 trial types, as with the navigation decision data. However, in this analysis, the regressors were event-related, and modelled the onset of the visual scene in the visual control component of each trial. In order to ensure that these regressors captured BOLD signal that was specifically related to the visual processing of each scene prior to the navigation decision period, we also included regressors for each of the 32 navigation decision periods, using a boxcar regressor in each case (exactly as used in the main analysis). Note that the inclusion of

these additional regressors in the GLM was necessary for this control analysis, but was not necessary in the main analysis, as visual scene information was always present within each navigation period in any case, and was not confounded with the key goal direction analyses. A visual control pattern similarity analysis was applied to the 32 voxel patterns representing the visual scene information on each trial. This analysis was a multiple binary regression analysis as described above, matched as closely as possible to the navigation decision analysis. The contrast regressor of interest specified the visual scene present on each trial, and three additional regressors-of-no-interest specified start location, goal location, and egocentric location for that trial. For a given ROI, beta parameters for the visual scene regressor were calculated for each participant, and compared against zero with a one-tailed t-test in order to determine the presence of purely visual scene information. This analysis was applied both to the left entorhinal/subicular ROI and also an anatomically defined ROI in bilateral extrastriate cortex. The scene information in both regions was compared with the geocentric direction information as measured in the main analysis in a 2x2 repeated measures ANOVA, with factors of region and information type.

There were two elements of the experimental design that provided sufficient temporal separation between the visual scene control and the navigation portions of each trial to make this control analysis feasible. First, each condition and trial-type occurs once in each of three functional sessions, and the first level GLM was set up to estimate a single response pattern expressed across all three sessions. Second, temporal jitter between the different components was a natural part of the design partly due to the self-paced nature of the task, but more importantly due to the randomized order of the different components. On each trial the order of the two control conditions was randomized, ensuring a degree of temporal jitter across trials. When measuring the response patterns over the three functional sessions, this allowed us to temporally separate the different signals of interest.

Egocentric control analysis

While investigating egocentric goal direction was not our principle aim in this study, we nevertheless included an additional egocentric control condition. In this condition, the same visual scene cue was presented as in the main navigation task, and a red dot was presented near this image. Participants simply had to indicate whether the dot was above, below, to the left, or to the right of the presented image. This task includes simple egocentric judgments that are not navigationally relevant. Thus, if a region contains information about this control task, then it is a more general egocentric representation rather than any specific representation related to navigation-guided behaviour. Egocentric control data were preprocessed using a first-level GLM to fit a regressor for each of the 32 trial types, as with the navigation decision data. However, in this analysis, the regressors were event-related, and modelled the onset of the red dot in the control component of each trial. Notably, unlike the visual control analysis described above, it was not necessary to additionally include the 32 navigation decision regressors. This is because the egocentric directions in the control task and the navigation task were both randomized, and therefore not confounded with one another across trials. An egocentric control RSA analysis was applied to the 32 voxel patterns representing egocentric direction onscreen on each trial. This analysis was a binary regression analysis contrasting trial pairs where the egocentric direction matched against those where the direction did not match. Within the precuneus ROI, beta parameters for this egocentric control regression were calculated for each participant, and compared against zero with a one-tailed t-test in order to determine the presence of simple egocentric direction information in the absence of navigation. This was directly contrasted with the beta parameters from the egocentric goal direction analysis with a two-tailed t-test.

Supplemental Results

Entorhinal lateralization analysis

The geocentric direction searchlight result found a result selectively within the left entorhinal/subicular region, suggesting that the head direction simulation effect may be left lateralized. In order to test this idea, we directly compared the geocentric direction information between the left and right entorhinal region, using anatomically defined regions of interest. These were manually delineated on the group average structural image using [S15] and [S16] for guidance. It should be noted that, as these ROIs were delineated on the group average structural image, not all anatomical landmarks were clearly visible. The ROI is therefore only an anatomical approximation of the entorhinal cortex (hence the term "entorhinal/subicular region"). Importantly, the same guidelines were used in both hemispheres, so this gives us an unbiased method of assessing any lateralized functionality. The analysis used the same pattern similarity multiple binary regressor as was used in the searchlight, but now specifically focusing on the two ROIs. This revealed that both hemispheres independently contain geocentric direction information (Left: $t(15) = 3.22$, $p = 0.0028$; Right: $t(15) = 1.89$, $p = 0.039$), and while the left does show a greater effect (consistent with the searchlight result), this difference was not statistically significant ($t(15) = 1.53$, $p = 0.15$). Thus we find no evidence for a hemispheric specialization, but instead find that both hemispheres independently process geocentric goal direction.

Precuneus lateralization analysis

While the original significant precuneus cluster is confined to the left hemisphere, the ROI used for all further analyses was based on a more lenient threshold, in order to ensure that sufficient voxels were included for the RSA analyses (see above). Notably, this larger cluster was bilateral in nature, and therefore all ROI-based results reported in the main text were based on bilateral

precuneus. Nevertheless, it is possible that these results were based largely on just the left hemisphere voxels. To investigate this, we formally tested for hemispheric specialization of the precuneus in our dataset. In order to do this, we divided the bilateral precuneus ROI into two at the midline, creating left and right precuneus ROIs. We applied the same multiple regression pattern similarity analysis to both independently, and found that both sides independently contain significant egocentric goal direction information (Left: $t(15) = 3.05$, $p = 0.004$; Right: $t(15) = 3.26$, $p = 0.0026$), with no significant difference in the strength of coding between the two ($t(15) = 0.0061$, $p = 0.995$). We therefore find no evidence for hemispheric specialization in egocentric goal direction coding in our data.

Start and goal location analyses

While the main aim of this study was to investigate direction representations, the experimental design also allowed us to investigate spatial representations, both for the start location and the goal location. We note, however, that for the latter analysis, goal location itself is completely confounded with the visual object used to cue this location on each trial. This cue is not a confound with respect to any of our primary effects of interest, but is for goal location. Therefore any results of this particular analysis should be interpreted with caution. No voxels survived whole-brain FWE correction for either the start or goal location analyses (see below for results using a more liberal threshold). Similarly, follow-up analyses in the functionally defined ROIs revealed no significant effect of either type of spatial information in either the entorhinal/subicular region or the precuneus. Thus, in the context of this study, we only find evidence relating to direction coding, not spatial location coding. This suggests that we were not able to detect grid cell representations, in contrast to previous investigations of the entorhinal region [S4, S5]. However, these previous studies sampled a much larger range of spatial locations in order to detect these representations, while our study was instead optimised for detecting

direction representations. In addition, in the current study, each trial required participants to represent both their own location within the environment, and the goal location, which could have added considerable noise to the self-location analysis.

Exploratory searchlight analyses

While we focus primarily on results that are statistically rigorous based on the relevant SVC region, it is also informative to ask whether any additional regions in the brain might show an equivalent effect that would otherwise not show up due to being outside our predicted region of interest. We therefore conducted additional, exploratory searchlight analyses of each condition in order to determine whether this might be the case. While any such results are not statistically robust, they are nevertheless useful for determining whether other regions outside the SVC regions might also be involved, and therefore targets for future research. In order to do this, we used the same initial voxel thresholding of pseudo-t > 3 in each case, and then where possible took the cluster extent threshold that was used to determine significance within the SVC of the relevant analysis. For the geocentric direction analysis, this threshold was k>88, and for the egocentric direction analysis it was k>49. For the start location analysis, no voxel within the SVC mask survived the initial pseudo-t threshold, and only a single cluster of three voxels survived this across the entire brain. It is clear, therefore that we find no evidence for spatial location representations within our data. The geocentric direction analysis similarly produces no additional regions outside the SVC mask with cluster size greater than the SVC threshold (k>88). However, the egocentric analysis revealed two addition clusters at this threshold, in the cerebellum (MNI coords: 0, -46, -41; cluster size k = 91) and the precentral gyrus (MNI coords: -56, -3, 34; cluster size k = 68). It is therefore possible that these additional regions may also contain information about egocentric goal direction. The (confounded) goal location analysis revealed two clusters at this liberal threshold, in bilateral

dorsolateral PFC (right MNI coord: 33, 9, 26; cluster size 254; left MNI coord: -26, 15, 27; cluster size 73).

Grey matter control analysis

To ensure that the reported results within the entorhinal/subicular region were not primarily driven by voxels falling within the white matter, we performed an additional control analysis restricted to grey matter voxels. During preprocessing, each participant's T1-weighted structural image was segmented into grey and white matter images, each of which has normalized values between 0 and 1, with higher values indicating higher likelihood of that voxel containing grey matter. These grey matter images were normalized into MNI space, as described above. We repeated the analysis of each condition (facing direction, goal direction, crossmatch) within the entorhinal/subicular ROI, but this time restricting the analysis to grey matter voxels, using subject-specific grey-matter masks (with a conservative grey threshold of 0.8). For all three conditions we again found a significant result within the entorhinal/subicular region (Facing: $t(15) = 3.48$, $p = 0.0017$; Goal: $t(15) = 1.88$, $p = 0.04$; Cross: $t(15) = 2.89$, $p = 0.0056$). It is very unlikely, therefore, that these effects are driven by activity taking place within the white matter.

Pattern construction analysis

In order to specifically test the hypothesis that a linear mixture of both facing and goal direction information is present on each trial, we applied a second analysis method based on "pattern construction" [S24–S27]. The logic of this analysis is simple: On a trial consisting of a North facing direction and an East goal direction (a N+E trial), we hypothesize that representations of both North and East will be active, and will be expressed as a linear combination of the voxel patterns for North and East. Within our data, we have a proportion of trials where the facing direction and goal direction are identical. On these trials, only one direction signal should be present within the

entorhinal/subicular region, and these we refer to as “pure” direction trials. We can take advantage of these trials in order to explicitly test the linear combination hypothesis. For the above example, we would do this by taking the average pattern across all “pure” North trials (both facing and goal direction are North), and the average pure East pattern, and summing these two patterns to create a prediction of what a N+E trial pattern should look like if the linear combination rule holds. We then simply correlate the actual N+E trial with our predicted N+E pattern to assess how well our prediction matches the data. We applied this logic to the voxel patterns within the entorhinal/subicular region of interest. The full analysis involved creating pure direction patterns for each of the four cardinal directions, as described above. From these we then constructed the full set of possible combined patterns by summing the different combinations of the pure directions. For each individual, we correlated the pattern for each trial against the predicted combined pattern for that trial (Note that these trials were completely independent of the data used to construct the pure and combined patterns). We also correlated the pattern for each trial against each of the non-predicted combined patterns for that trial, and averaged these correlation coefficients to provide a baseline null for that trial. The predicted and baseline correlation coefficients were averaged across all trials to form a summary for each participant. These were then statistically compared at the group level using a one-tailed paired t-test. This analysis revealed that the voxel pattern on each trial correlated significantly better with the predicted pattern than the unpredicted baseline patterns ($t(15) = 3.68$, $p = 0.0011$). This demonstrates that the constructed pattern, formed by a linear combination of both goal and facing direction, significantly predicts the pattern of voxel activity expressed on trials containing those two directions. Importantly, the predicted combined patterns also correlated with the trial voxel patterns significantly better than either the pure facing direction ($t(15) = 2.76$, $p = 0.015$; two-tailed t-test) or the pure goal direction pattern ($t(15) = 2.39$, $p = 0.03$; two-tailed t-test) for that trial, demonstrating that this result cannot be explained by either one of these alone –

instead, it is the linear combination of the two direction patterns that best explains the voxel pattern expressed on any given trial. These results provide further support for the conclusion that both facing and goal direction information are present within the entorhinal/subicular region.

Exploring the "pure" direction trials

As described above, we refer to those trials where both the facing direction and goal direction match as "pure" direction trials, as only one single geocentric direction representation should be active on these trials. We might expect that these trials should have a stronger representation of this direction compared to trials where two different directions are represented. In order to test this idea, we specifically assessed the correlation between the matching "pure" direction trials for each participant, and compared this against the facing direction and goal direction conditions (after excluding the pure direction trials). We found no evidence for a significantly greater level of similarity in the pure direction trials compared to either facing direction ($t(15)=-0.76$, $p=0.46$) or goal direction ($t(15)=-0.60$, $p=0.55$), as assessed with a two-tailed paired t-test. This therefore suggests that "pure" trials do not have a stronger directional representation. Indeed, we found that the similarity was somewhat lower in this condition than both the facing and goal direction conditions, although this was not significant in either case. We attribute this lower similarity score to the fact that the "pure" condition is based on just four correlation datapoints per subject, as opposed to 40 and 24 datapoints for facing and goal direction respectively. The small data sample for each subject is likely to have produced a much less reliable estimate of the true effect in the "pure" condition compared to either other condition. To examine the impact of sample size, we performed a bootstrap analysis using the facing direction correlation datapoints. The full sample includes 40 correlation datapoints per subject, which produced a significant effect compared to the null match condition ($t(15)=3.48$, $p=0.0017$). However, when we randomly sampled 4 of these correlation pairs per subject over 10,000 repetitions, the probability of detecting a significant

effect was found to be just 28%. When we increased the number of samples to 24, this probability increased to 94%, showing that sample size for each individual subject has a substantial impact on power at the group level. This therefore suggests that our "pure" direction analysis was substantially underpowered to detect our effect of interest, and this result should be interpreted accordingly.

Pattern construction visual control analysis

We applied a second method of checking whether the visual scene information could explain our data, this time using the pattern construction method (see above). The method was identical to that described above, with the exception that the "pure" direction patterns were now constructed from the visual scene data rather than the navigation data. In this case all visual scenes for each direction were averaged together to form each "pure" pattern. These were then used to construct each of the combined patterns that would be predicted if the neural patterns expressed on each navigation trial were the linear combination of a visual scene response in the facing direction, and an imagined visual scene in the goal direction (as opposed to representations of geocentric direction). Based on these predicted combined patterns, the pattern construction analysis was repeated. In this case, we found that the voxel pattern on each trial did not correlate significantly better with the predicted pattern than the unpredicted baseline patterns ($t(15)=-1.11$ $p=0.86$). Furthermore, the difference between predicted and unpredicted correlation was significantly greater in the navigation condition than this visual control condition ($t(15)=3.57$, $p=0.0028$). Overall, therefore, this analysis provides confirmatory evidence that our results cannot be explained on the basis of basic visual scene representations, but instead are likely to be based on representations of geocentric direction.

Associative learning control

The navigation task used in this experiment could not be solved by any simple paired association strategy between goal location and the correct direction (or scene), as the correct answer on each trial depended on the spatial relationship between the start location and the goal location. However, is it possible that, rather than a simple paired association, participants have encoded a more complex association between start location, goal location, and direction/scene? If participants were able to learn these associations through simple associative learning, then they would be able to solve the geocentric task without the need to use any spatial simulation. Critically, participants did not have the chance to explicitly learn all 32 of these sets of associative triads, as the learning of the virtual environment was accomplished through free exploration prior to scanning. However, 8 trials were explicitly presented, with feedback, as these "probe" trials were used to assess how well participants were learning the environment. While we note that these probe trials themselves could not have been solved through associative learning, it is in theory possible that exposure to these trials could have led to subsequent associative encoding of these 8 associative triads. To ensure that our results are not primarily due to these 8 trials, we ran an additional pattern similarity analysis controlling for these 8 probe trials. This was accomplished using the multiple binary regression approach described above, with the additional of one further regressor that indexed those trial pairs that included one or more probe trials. This additional regressor effectively captured variance associated with the probe trials in particular, and ensured that any remaining variance associated with geocentric direction could not be due to associatively learned probe trial information. This analysis revealed a significant effect for geocentric direction coding within the entorhinal/subicular region ROI ($t(15) = 3.47$, $p = 0.0017$), providing evidence that our results cannot be explained by associative learning.

Supplemental Discussion

Conjunctive grid cells

Given that previous studies have demonstrated the presence of grid cell representations within the entorhinal region, is it possible that our results could be due to grid cell rather than head-direction cell activity? Classical grid cells are not sensitive to direction information, and so it is not clear how they be responsible for our results. Conjunctive grid cells, on the other hand, do show modulation by heading direction. Thus, it is possible that the direction signal we detect in the entorhinal/subicular region is partially driven by conjunctive cell simulation of intended future vectors [S28]. However, entorhinal conjunctive cells are highly interconnected with head-direction cells, and indeed are assumed to receive their directional input from them [S6, S7]. It is therefore unlikely that conjunctive cells would be recruited for simulation without the connected head-direction cells also being recruited. Indeed, it is more likely that this simulation begins with head-direction simulation, which then drives conjunctive cell simulation [S29]. Notably, even a recent model of vector navigation based on conjunctive cell ensembles assumes a stable head-direction input to those conjunctive cells [S28]. This therefore implies that head-direction simulation must precede the conjunctive cell simulations of specific vectors for any direction other than current heading. We therefore argue that it is unlikely that conjunctive cell populations alone can explain our results. The most parsimonious explanation is that the goal direction representation is generated by head-direction simulation, but we cannot rule out that conjunctive cell simulation also contributes to this signal.

Attentional influence on entorhinal/subicular correlations

While we cannot completely rule out that differences in attention across subjects may have contributed to the correlation we found between entorhinal/subicular facing direction and task

performance, the fact that the results are specific to facing direction information, and not goal direction, makes this unlikely. Such global differences would be expected to produce a correlation in both facing and goal direction information.

Supplemental References

S1. Taube, J. S., Valerio, S., and Yoder, R. M. (2013). Is navigation in virtual reality with FMRI really navigation? J. Cogn. Neurosci. *25*, 1008–1019.

S2. Ekstrom, A. D., Kahana, M. J., Caplan, J. B., Fields, T. A., Isham, E. A., Newman, E. L., and Fried, I. (2003). Cellular networks underlying human spatial navigation. Nature *425*, 184–188.

S3. Hassabis, D., Chu, C., Rees, G., Weiskopf, N., Molyneux, P. D., and Maguire, E. A. (2009). Decoding neuronal ensembles in the human hippocampus. Curr. Biol. *19*, 546–554.

S4. Doeller, C. F., Barry, C., and Burgess, N. (2010). Evidence for grid cells in a human memory network. Nature *463*, 657–661.

S5. Jacobs, J., Weidemann, C. T., Miller, J. F., Solway, A., Burke, J. F., Wei, X.-X., Suthana, N., Sperling, M. R., Sharan, A. D., Fried, I., et al. (2013). Direct recordings of grid-like neuronal activity in human spatial navigation. Nat. Neurosci. *16*, 1188–1190.

S6. Sargolini, F., Fyhn, M., Hafting, T., McNaughton, B. L., Witter, M. P., Moser, M.-B., and Moser, E. I. (2006). Conjunctive representation of position, direction, and velocity in entorhinal cortex. Science *312*, 758–762.

S7. Whitlock, J. R., and Derdikman, D. (2012). Head direction maps remain stable despite grid map fragmentation. Front. Neural Circuits *6*, 9.

S8. Vass, L. K., and Epstein, R. A. (2013). Abstract representations of location and facing direction in the human brain. J. Neurosci. *33*, 6133–6142.

S9. Weiskopf, N., Hutton, C., Josephs, O., and Deichmann, R. (2006). Optimal EPI parameters for reduction of susceptibility-induced BOLD sensitivity losses: A whole-brain analysis at 3 T and 1.5 T. NeuroImage *33*, 493–504.

S10. Weiskopf, N., Hutton, C., Josephs, O., Turner, R., and Deichmann, R. (2007). Optimized EPI for fMRI studies of the orbitofrontal cortex: compensation of susceptibility-induced gradients in the readout direction. MAGMA *20*, 39–49.

S11. Carr, V. A., Rissman, J., and Wagner, A. D. (2010). Imaging the human medial temporal lobe with high-resolution fMRI. Neuron *65*, 298–308.

S12. Welvaert, M., and Rosseel, Y. (2013). On the Definition of Signal-To-Noise Ratio and Contrast-To-Noise Ratio for fMRI Data. PLoS ONE *8*, e77089.

S13. Misaki, M., Kim, Y., Bandettini, P. A., and Kriegeskorte, N. (2010). Comparison of multivariate classifiers and response normalizations for pattern-information fMRI. NeuroImage *53*, 103–118.

S14. Chadwick, M. J., Bonnici, H. M., and Maguire, E. A. (2012). Decoding information in the human hippocampus: A user's guide. Neuropsychologia *50*, 3107–3121.

S15. Duvernoy, H. M. (2005). The Human Hippocampus: Functional Anatomy, Vascularization and Serial Sections with MRI 3rd ed. (New York, USA: Springer).

S16. Insausti, R., Juottonen, K., Soininen, H., Insausti, A. M., Partanen, K., Vainio, P., Laakso, M. P., and Pitkänen, A. (1998). MR volumetric analysis of the human entorhinal, perirhinal, and temporopolar cortices. AJNR Am. J. Neuroradiol. *19*, 659–671.

S17. Vann, S. D., Aggleton, J. P., and Maguire, E. A. (2009). What does the retrosplenial cortex do? Nat. Rev. Neurosci. *10*, 792–802.

S18. O'Keefe, J., and Nadel, L. (1978). The Hippocampus as a Cognitive Map (Oxford University Press, USA).

S19. Eickhoff, S. B., Stephan, K. E., Mohlberg, H., Grefkes, C., Fink, G. R., Amunts, K., and Zilles, K. (2005). A new SPM toolbox for combining probabilistic cytoarchitectonic maps and functional imaging data. NeuroImage *25*, 1325–1335.

S20. Cavanna, A. E., and Trimble, M. R. (2006). The precuneus: a review of its functional anatomy and behavioural correlates. Brain J. Neurol. *129*, 564–583.

S21. Kriegeskorte, N., Goebel, R., and Bandettini, P. (2006). Information-based functional brain mapping. Proc. Natl. Acad. Sci. U. S. A. *103*, 3863–3868.

S22. Nichols, T. E., and Holmes, A. P. (2002). Nonparametric permutation tests for functional neuroimaging: a primer with examples. Hum. Brain Mapp. *15*, 1–25.

S23. Nili, H., Wingfield, C., Walther, A., Su, L., Marslen-Wilson, W., Kriegeskorte, N. (2014). A toolbox for representational similarity analysis. PLoS Comput. Biol. 10(4).

S24. Macevoy, S. P., and Epstein, R. A. (2009). Decoding the representation of multiple simultaneous objects in human occipitotemporal cortex. Curr. Biol. *19*, 943–947.

S25. Reddy, L., Kanwisher, N. G., and VanRullen, R. (2009). Attention and biased competition in multi-voxel object representations. Proc. Natl. Acad. Sci. U. S. A. *106*, 21447–21452.

S26. MacEvoy, S. P., and Epstein, R. A. (2011). Constructing scenes from objects in human occipitotemporal cortex. Nat. Neurosci. *14*, 1323–1329.

S27. Baeck, A., Wagemans, J., and Op de Beeck, H. P. (2013). The distributed representation of random and meaningful object pairs in human occipitotemporal cortex: the weighted average as a general rule. NeuroImage *70*, 37–47.

S28. Kubie, J. L., and Fenton, A. A. (2012). Linear look-ahead in conjunctive cells: an entorhinal mechanism for vector-based navigation. Front. Neural Circuits *6*, 20.

S29. Erdem, U. M., and Hasselmo, M. (2012). A goal-directed spatial navigation model using forward trajectory planning based on grid cells. Eur. J. Neurosci. *35*, 916–931.
